# No Association between Oxytocin Receptor (OXTR) Gene Polymorphisms and Experimentally Elicited Social Preferences

**DOI:** 10.1371/journal.pone.0011153

**Published:** 2010-06-16

**Authors:** Coren L. Apicella, David Cesarini, Magnus Johannesson, Christopher T. Dawes, Paul Lichtenstein, Björn Wallace, Jonathan Beauchamp, Lars Westberg

**Affiliations:** 1 Department of Health Care Policy, Harvard Medical School, Boston, Massachusetts, United States of America; 2 Department of Human Evolutionary Biology, Harvard University, Boston, Massachusetts, United States of America; 3 Department of Economics, Massachusetts Institute of Technology, Cambridge, Massachusetts, United States of America; 4 Department of Economics, Stockholm School of Economics, Stockholm, Sweden; 5 Department of Political Science, University of California San Diego, La Jolla, California, United States of America; 6 Department of Medical Epidemiology and Biostatistics, Karolinska Institutet, Stockholm, Sweden; 7 Department of Economics, Harvard University, Boston, Massachusetts, United States of America; 8 Department of Pharmacology, Institute of Neuroscience and Physiology, University of Gothenburg, Gothenburg, Sweden; Institute of Evolutionary Biology (CSIC-UPF), Spain

## Abstract

**Background:**

Oxytocin (OXT) has been implicated in a suite of complex social behaviors including observed choices in economic laboratory experiments. However, actual studies of associations between oxytocin receptor (OXTR) gene variants and experimentally elicited social preferences are rare.

**Methodology/Principal Findings:**

We test hypotheses of associations between social preferences, as measured by behavior in two economic games, and 9 single nucleotide polymorphisms (SNPs) of the OXTR gene in a sample of Swedish twins (n = 684). Two standard economic games, the dictator game and the trust game, both involving real monetary consequences, were used to elicit such preferences. After correction for multiple hypothesis testing, we found no significant associations between any of the 9 single nucleotide polymorphisms (SNPs) and behavior in either of the games.

**Conclusion:**

We were unable to replicate the most significant association reported in previous research between the amount donated in a dictator game and an *OXTR* genetic variant.

## Introduction

The field of behavioral economics has made significant strides during the last three decades in painting a panorama of the diversity of human economic behavior, a panorama which in several ways challenges the behavioral assumptions made in standard economic models [1]–[4]. Empirical research, mostly of experimental nature, has demonstrated that under a wide range of conditions, many people do not maximize their material payoffs, thereby exhibiting preferences that are sometimes characterized as “other-regarding” or “social” [5]. It is also now well known that there is ample individual heterogeneity in such other-regarding preferences [6]–[7]. Consequently, in recent years, the level of analysis in this literature has shifted from descriptive to explanatory, as researchers have increasingly sought to identify sources of individual differences. While economists have historically related behavioral variation to environmental variables, genetic sources of variation are currently being explored [8]. One promising area of research has been to examine the role of oxytocin (OXT) and its receptors on social behavior, including trust and generosity [9]–[10].

OXT is a nonapeptide synthesized primarily by the paraventricular (PVN) and supraoptic nuclei (SON) of the hypothalamus. Functioning as both a neurotransmitter and a neuromodulator, it exerts a wide range of effects both peripherally and centrally. The most notable peripheral targets of OXT include uterine and mammary tissue. OXT induces contractions during labor and milk “let down” during lactation. Recently, converging evidence from studies on human and non-human subjects has demonstrated central effects of OXT on a number of complex behaviors. For example, in studies of animals, including rodents, OXT has been shown to regulate maternal care, social recognition and other affiliative behaviors (for review, see 11). Overall, this line of research suggests that OXT might also modulate human social relationships [10]–[11].

Some evidence in favor of this proposition came from studies using paradigms from experimental economics. For example, one early study [12] related behavior in the trust game [13] to endogenous OXT levels. In the trust game, two players interact anonymously. The first player, the “trustor”, is given an endowment, and has the option of sending some fraction of this endowment to the second player, the “trustee”. The amount invested by the trustor is increased by some factor by the experimenter before entering the trustee's account. The trustee can choose to return some portion of the money to the subject or keep the money for themselves. The study found that trustees who receive signals of trust from trustors (e.g. money transfers) display higher levels of endogenous OXT compared to subjects who did not receive such signals. Trustees who display higher levels of OXT also return higher monetary amounts to their trustors [12]. The theoretical basis for this line of investigation is still unsettled, however, because peripheral oxytocin levels are only weakly correlated with the central OXT levels that were identified as important in the rodent work. Additionally, the reported associations in [12] were only marginally significant.

A number of research teams have subsequently documented effects of exogenously administrated OXT on a wide spectrum of social behaviors, including trust [14], generosity [15] and pair-bonding related phenotypes such as communication and behavior in a conflict discussion between couples [16]. Taken together, these results raise the possibility that OXT plays a role in behaviors associated with both trust and the reciprocation of trust (trustworthiness). Twin studies have reported that there is heritable variation in trust, trustworthiness [17] and generosity [18].

One candidate gene for genetic association studies involving social behavior, such as trust, is the OXTR gene. In humans, the OXTR gene, localized as a single copy on chromosome 3 [19], has been implicated in the development of autism, a phenotype characterized by deficits in social behavior and language development. Several independent studies identified the 3p25 region, where the OXTR gene is localized, as linked to autism [20]–[22]. Further studies have examined the association between single SNPs in the OXTR gene and autism [23]–[26], with mixed results. OXTR gene polymorphisms have also been associated with other social behaviors in humans such as empathy [27], prosocial decision making [28], attachment [29] and parenting [30]. A more recent study also identified a significant association between another marker (rs75775) and autism [31]. Finally, OXT and *OXTR* deficient mice display pervasive social deficits. For instance, *OXTR* knockout mice lack maternal nurturing [32], display increased aggression and are unable to recognize familiar conspecifics [32]–[33].

While the molecular evidence suggests that more extreme phenotypes are likely associated with the OXTR gene and that severe aberrations in the OXTR gene, such as deletions, are associated with major social deficits in rodents, it still remains unclear whether polymorphic differences in the OXTR gene can help explain normal variation in human social preferences. To our knowledge, only one study has examined the relationship between social preferences and the OXTR gene. Using the dictator game, a simple one-shot game in which a subject decides under conditions of anonymity how to divide an endowment between themselves and an unknown individual, researchers examined the association between 16 tagging SNPs across the entire OXTR gene and dictator game donations [28]. As a secondary measure of pro-social attitudes, the authors also administered a Social Values Orientation (SVO) task. In a first sample of 203 subjects, significant associations were found between the rs1042778, rs2268490 and rs237887 SNPs and both dictator game giving and behavior in the SVO task. The results for rs1042778 remained significant after Bonferroni correction. The association between dictator game giving and rs1042778 was successfully replicated in a second sample of 98 subjects [28], but the five other associations failed to replicate in that sample. While these results are interesting, the study represents only the first evidence indicating a role of the OXTR gene in social preferences and thus more work is needed before definite conclusions can be reached.

In this paper, we examine the relationships between nine *OXTR* polymorphisms (including rs1042778 and rs237887) and behavior elicited from two standard economic games, the dictator game and the trust game, in a sample of 685 individuals. The experiments were conducted with real monetary consequences, consistent with standard practice in experimental economics [34].

## Materials and Methods

### Subjects

The subjects were recruited in collaboration with the Swedish Twin Registry as part of a study on the heritability of experimentally elicited preferences. A detailed description of our sample, along with an analysis of non-response bias, is given in [18]. All of our invitees were same-sex twin pairs that had previously participated in the web-based survey STAGE, an acronym for “the Study of Twin Adults: Genes and Environment.” The subjects are born between 1959 and 1985. A total of 920 subjects participated in the experiments and out of these, 684 provided a biological specimen of sufficient quality to be used for genotyping. The final sample is comprised of 270 MZ twin pairs, 60 DZ twin pairs and 24 singletons.

### Genotyping

Nine SNPs in and up- and downstream of the OXTR gene were chosen for genotyping. One of the SNPs encodes an aminoacid substitution (rs4686302) and eight SNPs have been associated with autism and/or other social behaviors. See Table 1 for references and further information about the studied SNPs. Genotyping of SNPs rs75775, rs53576 and rs237887 was performed by KBioscience (http://www.kbioscience.co.uk) using the KASPar chemistry, which is a competitive allele specific PCR SNP genotyping system using FRET quencher cassette oligos (http://www.kbioscience.co.uk/genotyping/genotyping-chemistry.htm). The remaining six SNPs rs4686302, rs237897, rs2254298, rs2268493 and rs1042778 were genotyped using commercially available 5′ nuclease (TaqMan) assays on an ABI Prism 7900HT instrument (Applied Biosystems, Foster City, CA, USA).

**Table 1:** List of Analyzed SNPs.

Variable	Minor Allele	Minor Allele Frequency	# Obs	Hardy-Weinberg	SNP Position	Position	References/Proposed Association
rs75775	T	0.169	645	0.785	8795732	5′	Autism [31]
rs4686302	T	0.135	676	0.858	8784222	exon 3	nonsynonymous SNP A218T
rs237897	A	0.405	660	0.414	8783285	intron 3	Dictator game giving [28]; Autism/IQ [24]
rs53576	A	0.347	645	0.328	8779371	intron 3	Unipolar depression and adult separation anxiety [29]; Maternal sensitivity [30]; Autism [23]
rs2254298	A	0.088	674	0.366	8777228	intron 3	Autism [23], [36]; Unipolar depression [29]; Adult separation anxiety [29]
rs2268493	C	0.348	664	0.908	8775840	intron 3	Autism [26]
rs237887	G	0.380	665	0.242	8772042	intron 3	Dictator game giving [28]; Communication & daily living [24];
rs1042778	T	0.412	642	0.630	8769545	exon 4/3utr	Dictator game giving [28]; Autism/IQ [24], [25]
rs7632287	A	0.264	663	0.716	8766446	3′	Autism [25]

Notes: Tests of Hardy-Weinberg conducted using likelihood ratio tests using only a sample of genetically unrelated individuals (one twin from each pair was randomly selected if genotypic data was available for both twins).

Table 1 reports summary information for the nine SNPs in our sample, along with information on the minor allele frequencies, the number of individuals which could be genotyped at each locus and p-values for the tests of Hardy-Weinberg equilibrium, which were conducted using Stata's genhwi program [35]. We cannot reject the null hypothesis of Hardy-Weinberg equilibrium at conventional levels of significance for any of the SNPs. This suggests that our study population is not too far from genetic equilibrium and that it is unlikely that there were systematic genotyping errors. The final column gives an overview of previously proposed phenotypic associations for each SNP [23]–[26], [28]–[31], [36].

### Experimental Procedures

Upon arrival, subjects were instructed not to talk to each other during the experiment and to raise their hands if they had any questions (such questions were rare and were answered in private). They were also told about the strong norm against deception in experimental work in economics. Twins in the same twin pair were always required to take part in the same experimental session, thus ruling out the possibility of communication about the experiments. The instructions also made it clear that subjects would never be paired with their twin sibling when playing the experimental games, but would rather be paired against some other anonymous participant. Below, we describe how we administered the dictator and the trust games.

Dictator Game – To measure preferences for giving, we used a modified dictator game. In a standard dictator game [37], the “dictator” decides how to split some endowment between herself and another person [1]. A variant of this approach first used by Eckel and Grossman [38] is that the subject decides how to allocate a sum of money between herself and a charity. In the present study subjects decided how to allocate SEK 100 (about $15) between themselves and a charity called “Stadsmissionen”. Stadsmissionen's work is predominantly focused on helping the homeless in Sweden. Our measure of giving is simply the amount of money donated by the dictator to charity.

Trust Game – We administered a standard trust game [13] in which subjects first played the role of trustor and then trustee albeit with a different anonymous partner. In the first stage, subjects were given an endowment of SEK 50, of which they could transfer any amount to the trustee in multiples of 10. Both players were informed that any amount transferred would be multiplied by 3 before being sent to the trustee. The trustee was then given the option of reciprocating by sending any fraction of the transferred amount back to the trustor. To elicit the trustworthiness of the trustee, we used the strategy method [1]. That is, subjects indicated how they would react to any possible amount sent prior to observing trustor behavior. The actual investment decision was then realized, and subjects were paid in accordance with the decision of the trustee at that node. Our measure of trust is the amount of money transferred in the role of trustor. Our measure of trustworthiness is the average fraction returned at the five decision nodes.

PC – Finally, because we were concerned that the elicitation of preferences using a one-shot game is quite noisy, we applied principal components analysis to the three variables and used the first principal component as a fourth measure of “social” preferences. The first principal component correlates moderately with dictator game giving (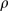 = 0.684), trust (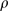 = 0.560) and trustworthiness (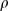 = 0.717).

### Ethics

This study was conducted according to the principles expressed in the Declaration of Helsinki. The study was approved by the Regional Ethical Review Board of Ethics in Stockholm. All subjects gave written informed consent.

### Statistical Methods

We used linear regression analysis to test for association, see e.g. chapter 15 in [39]. For each of our four outcome variables, (dictator game giving, trust, trustworthiness and their first principal component), we ran individual regressions on one SNP at a time, controlling for age and sex. Our baseline specification is an additive model of the form,
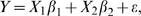
where 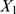 is a matrix with a constant, age and sex and 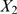 is the individual's genotypic score, taking the value −1 if the individual is homozygous for the major allele, 0 if the individual is heterozygous and 1 if the individual is homozygous for the minor allele. The specification is additive, because the conditional expectation function is linear in the number of alleles. Since there are nine OXTR gene SNPs and four outcome variables, we ran a total of 36 regressions.

The additive model with controls for age and sex is a natural baseline model to consider, but it does not admit differential genetic effects by sex. Israel et al [28] reported some results suggestive of sex specific effects and there is also evidence of sex differences in the effects of OXT from both animal studies [40] and human studies [41]. To examine this, we also estimated a modified additive model which allows for differences in genetic effects between men and women,
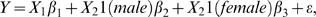
where 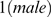 is an indicator variable taking the value 1 if subject is male and 0 otherwise and 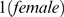 is defined analogously. In this specification, 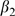 is the average change in phenotype associated with having an additional minor allele in men, holding the remaining covariates constant. The corresponding coefficient in women is 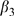.

As a final robustness check, we also estimated non-additive (dominance) models. We augmented the model with an additional dummy variable which takes the value 1 if the individual is heterozygous at that locus, thus allowing for the possibility that the mean phenotypic value of the heterozygotes is not the midpoint of the phenotypes of the two homozygotes.

Since we are analyzing twin pairs, the error terms are non-independent for observations within the same family. Let 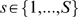 index the singletons, 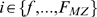 the MZ families, 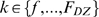 the DZ families, and 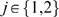 the individuals within a family. Let 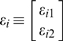 and define 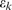 analogously. Then we can define 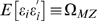 and 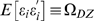. Without loss of generality, order the observations by family size (putting the singletons before twin pairs), zygosity and family ID. If the errors are homoscedastic and observations from different families are independent, then
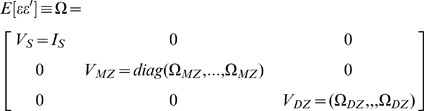
The diagonal entries of this matrix 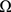 are estimated as the sample variance of the regression residuals. The off-diagonal entries in the 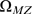 (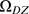) matrix are similarly estimated as the sample covariance of the regression residuals between MZ (DZ) twins. From this, we can construct 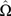 and plug it into the standard estimator of the variance covariance matrix of the regression coefficients, which is how we obtain the standard errors of our estimated coefficients.

We use the genetic markers in our dataset to establish zygosity of the twin pairs. Specifically, twin pairs who differ in the number of minor alleles on at least one locus are assumed to be DZ and all other twins are classified as MZ. In analyses not shown here, we verified that the standard errors are substantively identical if we use the Twin Registry's algorithm for classifying zygosity rather than the genetic data.

## Results

We begin with some summary statistics. Table 2 reports, separately for MZ and DZ twins, summary statistics for the outcome variables, dictator game giving, trust, trustworthiness and the principal component of these variables. We also report the age of our respondents and their educational attainment, in years. The sample is predominantly female and the average participant has about two years of college.

**Table 2:** Summary Statistics for the Sample.

	MZ Twins			DZ Twins		
Variable	Obs	Mean	Std. Dev.	Obs	Mean	Std. Dev
Dictator Game Giving	546	52.91	37.35	138	60.84	37.49
Trust Invested	545	38.73	14.03	138	40.79	12.56
Trust Fraction Returned	543	36.68	15.93	138	39.29	15.20
1 if female	546	0.80	0.40	138	0.74	0.44
Year of Birth	546	1973	7.47	138	1972	7.89
Educational Attainment	546	14.00	2.27	137	13.88	2.41

Notes: Years of education estimated from categorical variable produced by Statistics Sweden (with seven categories ranging from middle school to PhD).

Results from the additive specification are reported in Table 3. None of the individual SNPs are significant at the five percent level and only three SNP (rs75775, rs2268493 and rs1042778) are significant at the ten percent level for one of the outcome variables, namely trust. Since none of the nominal, uncorrected, p-values are below five percent it obviously follows that none of the markers are statistically significant after correction for multiple hypothesis testing.

**Table 3:** Regression Results: Additive Model.

	Dictator Game	Trust	Trustworthiness	PC
Variable	Coefficient (s.e.)	Coefficient (s.e.)	Coefficient (s.e.)	Coefficient (s.e.)
rs75775	0.56	2.10*	1.11	0.13
	(3.01)	1.09	(1.22)	(0.09)
	p = 0.852	p = 0.055	p = 0.363	p = 0.162
rs4686302	−1.05	−0.96	−1.06	−0.09
	(3.23)	(1.18)	(1.32)	(0.10)
	p = 0.745	p = 0.413	p = 0.422	p = 0.335
rs237897	3.21	−0.89	−0.06	0.02
	(2.34)	(0.86)	(0.95)	(0.07)
	p = 0.171	p = 0.300	p = 0.947	p = 0.829
rs53576	2.99	−1.41	0.13	0.00
	(2.37)	(0.87)	(0.98)	(0.07)
	p = 0.207	p = 0.103	p = 0.893	p = 0.984
rs2254298	0.94	−1.66	1.99	0.03
	(3.86)	(1.40)	(1.58)	(0.12)
	p = 0.808	p = 0.236	p = 0.207	p = 0.798
rs2268493	0.52	−1.40*	−1.01	−0.08
	(2.31)	(0.85)	(0.95)	(0.07)
	p = 0.821	p = 0.098	p = 0.291	p = 0.242
rs237887	2.16	−1.46*	0.99	0.01
	(2.23)	(0.80)	(0.91)	(0.07)
	p = 0.333	p = 0.069	p = 0.276	p = 0.828
rs1042778	1.56	0.12	0.02	0.04
	(2.27)	(0.84)	(0.95)	(0.07)
	p = 0.493	p = 0.891	p = 0.978	p = 0.614
rs7632287	−0.76	−0.42	.27	−.01
	(2.53)	(0.94)	(1.04)	(0.08)
	p = 0.764	p = 0.652	p = 0.796	p = 0.872

Notes: This table reports regression coefficients from the additive model, estimated separately for each individual SNP. All regressions include age and sex controls. One star (*) denotes statistical significance at the ten percent level (three coefficient estimates are statistically significant at the ten percent level, and none is significant at the five percent level).

Table 4 gives the results from the additive model with sex specific effects. The most significant association is observed between rs75775 and dictator game giving in men. Each additional T allele is associated with a 16 SEK decline in the donation to charity and the p-value of the regression coefficient, unadjusted for multiple hypothesis testing, is 0.008. However, given the large number of hypotheses tested, the finding could easily be due to chance. Indeed, the Bonferroni corrected p-value exceeds even the most liberal thresholds. Yet, given that rs75775 has previously been implicated in autism [31], a phenotype related to various aspects of social behavior, it may be advisable to include this marker in future studies of the genetic basis of social preferences.

**Table 4:** Regression Results: Additive Model with Sex Specific Effects.

	Dictator Game		Trust		Trustworthiness		PC	
Variable	Coefficient (s.e.)		Coefficient (s.e.)		Coefficient (s.e.)		Coefficient (s.e.)	
	Women	Men	Women	Men	Women	Men	Women	Men
rs75775	5.58*	−16.32***	2.55**	0.60	1.89	−1.49	0.25**	−0.30
	(3.38)	(6.19)	(1.25)	(2.26)	(1.39)	(2.52)	(0.10)	(0.19)
	p = 0.098	p = 0.008	p = 0.041	p = 0.790	p = 0.174	p = 0.554	p = 0.013	p = 0.108
rs4686302	1.70	−11.16	−1.16	−0.23	−0.52	−3.02	−0.04	−0.31
	(3.63)	(6.94)	(1.33)	(2.51)	(1.49)	(2.81)	(0.11)	(0.21)
	p = 0.639	p = 0.107	p = 0.381	p = 0.928	p = 0.724	p = 0.283	p = 0.739	p = 0.143
rs237897	2.77	4.91	−0.44	−2.66	−0.50	1.64	0.01	0.05
	(2.62)	(5.15)	(0.96)	(1.89)	(1.07)	(2.08)	(0.08)	(0.16)
	p = 0.291	p = 0.340	p = 0.65	p = 0.158	p = 0.638	p = 0.432	p = 0.931	p = 0.757
rs53576	2.44	5.20	−0.91	−3.41*	0.10	0.27	0.01	−0.03
	(2.66)	(5.25)	(0.97)	(1.92)	(1.10)	(2.17)	(0.08)	(0.16)
	p = 0.359	p = 0.322	p = 0.349	p = 0.075	p = 0.931	p = 0.901	p = 0.912	p = 0.859
rs2254298	−0.23	7.28	−1.52	−2.38	2.57	−1.13	0.04	−0.01
	(4.21)	(9.65)	(1.52)	(3.55)	(1.72)	(3.99)	(0.13)	(0.29)
	p = 0.956	p = 0.451	p = 0.316	p = 0.503	p = 0.134	p = 0.776	p = 0.766	p = 0.967
rs2268493	2.41	−6.38	−1.82*	0.14	−1.42	0.48	−0.08	−0.08
	(2.60)	(4.99)	(0.96)	(1.82)	(1.08)	(2.05)	(.08)	(.15)
	p = 0.353	p = 0.202	p = 0.057	p = 0.940	p = 0.189	p = 0.815	p = 0.294	p = 0.604
rs237887	1.78	3.44	−1.64*	−0.84	1.56	−0.93	0.02	−0.01
	(2.54)	(4.69)	(0.91)	(1.69)	(1.03)	(1.91)	(0.08)	(0.14)
	p = 0.483	p = 0.463	p = 0.073	p = 0.618	p = 0.132	p = 0.627	p = 0.771	p = 0.937
rs1042778	3.04	−4.61	0.76	−2.55	−0.24	1.10	0.07	−0.12
	(2.53)	(5.09)	(0.94)	(1.88)	(1.06)	(2.13)	(0.08)	(0.16)
	p = 0.228	p = 0.365	p = 0.416	p = 0.176	p = 0.822	p = 0.605	p = 0.345	p = 0.437
rs7632287	−1.63	2.24	−0.45	−0.31	−0.38	2.50	−0.05	0.13
	(2.86)	(5.23)	(1.06)	(1.93)	(1.18)	(2.14)	(0.09)	(0.16)
	p = 0.570	p = 0.669	p = 0.669	p = 0.871	p = 0.747	p = 0.243	p = 0.548	p = 0.428

Notes: This table reports regression coefficients from the additive model, estimated separately for each individual SNP and allowing different coefficients in men and women. All regressions include age and sex controls. Three stars (***) denote statistical significance at the one percent level, two stars (**) denote statistical significance at the five percent level and one star (*) denotes statistical significance at the ten percent level.

Results from the non-additive specification are given in Table 5. The first column shows the estimated regression coefficient on the additive component, and the second column shows the estimated deviation of the heterozygote from the mean of the two homozygotes. The third column shows an F-test for the joint significance of these two coefficients. For trust, two of the SNPs - rs75775 and rs2254298 - are significant at the five percent level, but since a total of 36 hypotheses were tested this result does not survive multiple hypothesis correction and must hence be approached with caution.

**Table 5:** Regression Results: Non-Additive Model.

	Dictator Game			Trust			Trustworthiness			PC		
Variable	Add	Dom	p-value	Add	Dom	p-value	Add	Dom	p-value	Add	Dom	p-value
rs75775	−1.93	3.73	0.794	−0.71	4.21*	0.020	1.93	−1.23	0.572	0.02	0.16	0.245
	(4.85)	(5.70)		(1.73)	(2.04)		(1.96)	(2.30)		(0.14)	(0.17)	
rs4686302	−4.53	4.96	0.710	−0.62	−0.49	0.699	−4.54**	4.98*	0.120	−0.28	0.26	0.265
	(5.60)	(6.48)		(2.02)	(2.33)		(2.26)	(2.61)		(0.17)	(0.19)	
rs237897	3.39	−0.89	0.377	−1.02	0.65	0.505	−0.46	1.99	0.328	−0.00	0.08	0.719
	(2.43)	(3.30)		(.89)	(1.21)		(0.99)	(1.33)		(0.07)	(0.10)	
rs53576	2.68	.97	0.436	−1.21	−0.61	0.239	−0.37	1.55	0.555	−0.01	0.05	0.914
	(2.62)	(3.51)		(.95)	(1.27)		(1.08)	(1.44)		(0.08)	(0.11)	
rs2254298	9.86	−12.05	0.354	3.15	−6.50	0.048	4.47	−3.36	0.290	0.45**	−0.57**	0.085
	(7.42)	(8.44)		(2.65)	(3.03)		(3.00)	(3.42)		(0.22)	(0.25)	
rs2268493	2.49	−6.61	0.155	−0.85	−1.86	0.086	−.99	−0.07	0.573	−0.03	−0.18*	0.124
	(2.50)	(3.44)		(0.92)	(1.24)		(1.04)	(1.40)		(0.08)	(0.10)	
rs237887	2.07	0.43	0.620	−1.27	−.90	0.145	1.07	−0.40	0.529	0.02	−0.04	0.914
	(2.33)	(3.33)		(0.84)	(1.19)		(0.95)	(1.35)		(0.07)	(0.10)	
rs1042778	2.02	−2.72	0.561	0.31	−1.15	0.627	−0.21	1.31	0.630	0.04	−0.04	0.816
	(2.34)	(3.28)		(0.86)	(1.20)		(.98)	(1.36)		(0.07)	(0.10)	
rs7632287	−1.55	1.75	0.870	−1.17	1.66	0.483	−0.19	1.01	0.801	−0.07	0.13	0.546
	(3.12)	(4.00)		(1.15)	(1.48)		(1.28)	(1.64)		(0.10)	(0.12)	

Notes: This table reports regression coefficients from the non-additive model, estimated separately for each individual SNP. All regressions include age and sex controls. The reported p-values are for the F-test of the joint hypothesis that the additive and dominance coefficients are both equal to zero.

Finally, we also estimated the non-additive models allowing different coefficients in men and women and then tested the joint significance of either the male coeffients or the female coefficients. This entails a de facto doubling of the number of hypotheses being tested. The F-test of joint significance failed to reject the null hypothesis at the one percent level in all 72 cases, which is consistent with the overall pattern of null results. In a post hoc analysis we also verified that the replication failure does not appear to stem from the fact that Israel et al. [28] estimated dominant models, rather than the additive and dominance models considered here. Estimating dominant models using regression analysis, neither rs1042778 nor rs237887 – the two most promising SNPs reported by Israel et al. [28] – were significant at the ten percent level for any of the three phenotypes or their principal component.

## Discussion

Advances in human genetics have provided a large number of opportunities for studies of genetic association, but also a growing recognition that many published associations fail to replicate [42]–[43]. It is well known that, as an empirical matter, the problem of replication is especially acute in cases where the original association was based on a small sample [43], [44].

In the present study, we failed to detect any significant associations between 9 SNPs of the OXTR gene and social preferences as elicited from two standard economic games. Specifically, after correction for multiple hypothesis testing, we did not find any significant associations with allocations of funds in the dictator game or with trust or trustworthiness. The results reported here thus stand in contrast to a recent study which reported associations between variants of the OXTR gene and behavior in the dictator game as well as in the Social Value Orientation task [28]. For both the dictator game and the SVO task, Israel et al [28] reported a significant association with rs1042778 and some suggestive associations with two additional SNPs, namely rs2268490, and rs237887; they also replicated the association between dictator game giving and rs1042778 in an independent sample. The five other associations failed to replicate in the second sample. We do not find any strong evidence for a role for either rs1042778 or rs237887 as a source of individual differences in dictator game giving, trust or trustworthiness in either an additive model or a dominance model. The other suggestive SNP reported by Israel et al [28], rs2268490, was not typed in our sample. However, using the founders of the CEU population in Hapmap to obtain linkage disequilibrium (LD) statistics we found that one of our SNPs, rs2254298, is in moderate LD with rs2268490 (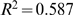), rendering it less likely that the failure to replicate is due to incomplete coverage. Our most significant association is between the SNP rs75775 located upstream of *OXTR* and pro-social behavior in men. We are reluctant to attach too much significance to this finding because it could easily be due to sampling variation, but we do note that two recent studies reported that this and another SNP in the 5′-region of *OXTR* were associated with autism [31], [45]. Perhaps future studies of social behaviors should include SNPs covering the upstream region of *OXTR*.

Non-reproducibility does not necessarily demonstrate that the original association reported was spurious. True associations may not replicate across different data sets for a number of reasons, including insufficient statistical power. Indeed, while our sample is larger than previous studies that have examined associations between experimentally elicited preferences and genetic variants, our power to detect weak genetic effects is still limited. This, coupled with the fact that original studies tend to overestimate effect sizes because of a winner's curse effect [46], could explain our failure to replicate. Additionally, the Israeli population studied by Israel et al. [28] is both genetically and environmentally distinct from our Swedish sample and this may also explain the difference in results. For example, the discrepancy in results may be due to genuine treatment effect heterogeneity, meaning that the variant they identified has a causal effect in some environments but not others. Alternatively, different patterns of linkage disequilibrium between the SNP and the true causal variant in different populations could explain the difference in results [47]–[49].

Given that our research design only allows us to statistically reject moderate to large effect sizes, the results reported here are not inconsistent with the results of hormonal association studies involving OXT in trust and generosity and do not necessarily rule out a role for *OXTR* polymorphisms in explaining phenotypic variation. An important implication of our results, however, is that sample sizes an order of magnitude greater than those used here will probably be necessary for understanding the pathways from causal variants to complex outcomes. This conclusion is of course in line with a growing consensus in molecular genetics that common genetic variants with large effects on complex outcome variables are unlikely to exist. While failed replications such as the one presented here are common and cautionary, they should not discourage further research in this promising field.
